# Draft assembly and annotation of the Cuban crocodile (*Crocodylus rhombifer*) genome

**DOI:** 10.1186/s12863-024-01240-y

**Published:** 2024-06-06

**Authors:** Robert W. Meredith, Yoamel Milián-García, John Gatesy, Michael A. Russello, George Amato

**Affiliations:** 1 Department of Biology, Montclair State University, Montclair, NJ USA; 2 Institute for Comparative Genomics, American Museum of Natural History, Central Park West at 79th Street, New York, NY 10024 USA; 3 Department of Integrative Biology, University of Guelph, Guelph, ON N1G 2W1 Canada; 4 Department of Biology, University of British Columbia, 3247 University Way, Kelowna, BC V1V 1V7 Canada

**Keywords:** Cuban crocodile, *Crocodylus rhombifer*, Critically Endangered, Conservation, Genome assembly, Genome annotation, Genomics

## Abstract

**Objectives:**

The new data provide an important genomic resource for the Critically Endangered Cuban crocodile (*Crocodylus rhombifer*). Cuban crocodiles are restricted to the Zapata Swamp in southern Matanzas Province, Cuba, and readily hybridize with the widespread American crocodile (*Crocodylus acutus*) in areas of sympatry. The reported de novo assembly will contribute to studies of crocodylian evolutionary history and provide a resource for informing Cuban crocodile conservation.

**Data description:**

The final 2.2 Gb draft genome for *C. rhombifer* consists of 41,387 scaffolds (contigs: N50 = 104.67 Kb; scaffold: N50-518.55 Kb). Benchmarking Universal Single-Copy Orthologs (BUSCO) identified 92.3% of the 3,354 genes in the vertebrata_odb10 database. Approximately 42% of the genome (960Mbp) comprises repeat elements. We predicted 30,138 unique protein-coding sequences (17,737 unique genes) in the genome assembly. Functional annotation found the top Gene Ontology annotations for Biological Processes, Molecular Function, and Cellular Component were regulation, protein, and intracellular, respectively. This assembly will support future macroevolutionary, conservation, and molecular studies of the Cuban crocodile.

## Objective

Crocodiles (Crocodylidae) are large semi-aquatic predators found throughout the tropics of Asia, Australia, Africa, and the Americas. Of the three extant genera (*Crocodylus*, *Osteolaemus*, and *Mecistops*) within Crocodylidae, *Crocodylus* is the largest, comprising 13 currently recognized species. The Cuban crocodile (*Crocodylus rhombifer*) is a Critically Endangered [1] island endemic, currently restricted to the smallest range of any extant member of the genus [2]. Fossil evidence suggests that it may be a Pleistocene relict formerly much more widespread in the Caribbean and Bahama islands [3, 4]. Now only found naturally in the Zapata Swamp in southern Matanzas Province, Cuba, *C. rhombifer* is restricted to the unique freshwater ecosystem characteristic of the Zapata peninsula. A long history of over-harvesting and land conversion continues to threaten this declining population. In addition, hybridization with the widespread American crocodile (*Crocodylus acutus*) in areas of sympatry may be an additional anthropogenic threat exacerbated by freshwater management and habitat modification activities [2].

A number of distinguishing morphological and behavioral traits have been described for this species [5, 6]. These include prominent cranial ‘horns’, heavy-scaled and colorful skin, robust skull structures, adaptations for a more terrestrial lifestyle, and aggressive, intelligent hunting strategies [5, 7]. Previous phylogenetic and phylogenomic studies are ambiguous about the exact phylogenetic placement of *C. rhombifer* within the monophyletic Neotropical *Crocodylus* radiation [2, 8–10]. Sequencing of whole genomes provides the best opportunity to test hypotheses concerning the biogeographic history and the evolution of novel morphological and behavioral traits. Such information may further offer insights into conservation threats and opportunities for this enigmatic species. Presented here is the first genome assembly for the Cuban crocodile.

## Data description

For a detailed description of all methods see Table 1, Data file 1. High molecular weight DNA was extracted from a non-hybrid Cuban crocodile ( [2]; Table 1, Data file 2) using the QIAGEN® MagAttract HMW DNA Kit. 10X Genomics Chromium Genome library preparation and sequencing was performed at the New York Genome Center. The libraries were 150 bp paired-end sequenced on an Illumina HiSeqX machine (1,717.59 million reads at ~ 65X coverage; mean read length of 138.5 bp; Table 1, Data file 3).

Two assemblies were performed. First, the linked reads were assembled into 41,387 scaffolds (contigs: N50 = 104.67 Kb; scaffolds: N50 = 518.55 Kb) using the Supernova assembler (v 2.1.1; [11]). The estimated genome size was 2.61 GB, and the assembly size was 2.20 Gb. The Supernova scaffolds were screened for contaminants via the NCBI Foreign Contamination Screen (https://github.com/ncbi/fcs), resulting in 39,474 scaffolds. For the second build, the Supernova assembly was run through RagTag [12] with the *Crocodylus porosus* genome (Cpor 3.0; [13]) as a reference. The RagTag assembly placed 19,264 contigs (25,753 scaffolds; N50 = 6,528.07 Kb: Table 1, Data file 3).

Completeness and quality of the two *C. rhombifer* genomic builds were assessed by Benchmarking Universal Single-Copy Orthologs (BUSCO v5.1.2; [14]) using the vertebrata_odb10 database (3,354 markers) and compared to published Crocodylia genomes (Table 1, Data file 4). The Supernova build had 91.3% of the BUSCO genes complete (single and duplicate), 5.1% fragmented (171 genes), and 2.6% missing (85 genes). The RagTag build had 95% of the BUSCO genes complete (single and duplicate), 3.0% fragmented (102 genes), and 2.0% missing (67 genes) (Table 1, Data file 4).

RepeatModeler and RepeatMasker [15] and Earl Gray [16, 17] identified ~ 1000Mbp of the builds as interspersed repeat elements. Retroelements (17–18%) and Unclassified (16–18%) were the most common (Table 1, Data file 5, 6). Protein sequences were predicted using two ab initio methodologies BRAKER2 [18–23] and MetaEuk [24]. This resulted in 30,138 unique protein-coding sequences (17,737 unique genes) (Table 1, Data file 7). PANNZER2 [25] was used for functional annotation. The top gene ontology annotations for biological processes, molecular function, and cellular component were regulation, protein, and intracellular, respectively (Table 1, Data files 8, 9). Orthofinder [26, 27] was used to perform comparative genomic analyses between all published crocodylian genomes. A total of 175,928 genes were compared among the five species. Of these, 93.5% were placed into 26,551 orthogroups, with 0.6% of genes in species-specific orthogroups (Table 1, Data files 10, 11).

BUSCO Phylogenomics [28] identified and aligned 1,912 single-copy BUSCO genes present in 12 taxa (five Crocodylia; seven outgroups). IQ-TREE inferred the maximum-likelihood concatenated protein tree with bootstrap support [29–31]. All recovered nodes had 100% bootstrap support (Table 1, Data file 12, 13).

**Table 1:** Overview of data files/data sets

Label	Name of data file/data set	File types (file extension)	Data repository and identifier (DOI or accession number)
*Data file 1*	Table 1, *Data file 1 Detailed description of the methodology*	*Microsoft Word (.docx)*	Figshare: 10.6084/m9.figshare.25388386 [32]
*Data file 2*	Table 1, *Data file 2 Photos of Cuban crocodiles*	*Microsoft Word (.docx)*	Figshare: 10.6084/m9.figshare.25388386 [32]
*Data file 3*	Table 1, *Data file 3_C.rhombifer10X_Assembly_statistics*	*Spreadsheet (.xlsx)*	Figshare: 10.6084/m9.figshare.25388386 [32]
*Data file 4*	Table 1, *Data file 4 BUSCO Comparisons*	*Microsoft Word (.docx)*	Figshare: 10.6084/m9.figshare.25388386 [32]
*Data file 5*	Table 1, *Data file 5 Interspersed Repeat landscape*	*Microsoft Word (.docx)*	Figshare: 10.6084/m9.figshare.25388386 [32]
*Data file 6*	Table 1, *Data file 6 Percentages of repeat elements*	*Microsoft Word (.docx)*	Figshare: 10.6084/m9.figshare.25388386 [32]
*Data file 7*	Table 1, *Data file 7 C.rhombifer10X_Pannzer*	*Portable document format (.pdf)*	Figshare: 10.6084/m9.figshare.25388386 [32]
*Data file 8*	Table 1, *Data file 8 Venn diagram*	*Microsoft Word (.docx)*	Figshare: 10.6084/m9.figshare.25388386 [32]
*Data file 9*	Table 1, *Data file 9 GO_counts_Table*	*Spreadsheet (.xlsx)*	Figshare: 10.6084/m9.figshare.25388386 [32]
*Data file 10*	Table 1, *Data file 10_Orthofinder_Results_Crocs_Only*	*Spreadsheet (.xlsx)*	Figshare: 10.6084/m9.figshare.25388386 [32]
*Data file 11*	Table 1, *Data file 11_Statistics_PerSpecies*	*Spreadsheet (.xlsx)*	Figshare: 10.6084/m9.figshare.25388386 [32]
*Data file 12*	Table 1, *Data file 12 Concordance factor statistics*	*Spreadsheet (.xlsx)*	Figshare: 10.6084/m9.figshare.25388386 [32]
*Data file 13*	Table 1, *Data file 13 Phylogeny*	*Microsoft Word (.docx)*	Figshare: 10.6084/m9.figshare.25388386 [32]
*Data set 1*	*Sequencing reads of C. rhombifer genomic DNA*	*Fastq files (.fq.gz)*	NCBI SRA Database: SAMN36978604 https://identifiers.org/ncbi/bioproject:PRJNA1005273 [33]
*Data set 2*	*Genomic Assembly of C. rhombifer*	*Fasta file (.fa)*	NCBI GenBank Database: JAVSML000000000 https://identifiers.org/nucleotide:JAVSML000000000 [34]

### Limitations

The draft genome was generated using short-read shotgun sequencing via 10X genomics for a scale sample. As a result, the assembly is somewhat fragmented and smaller than the genome size estimate. The Cuban crocodile is naturally restricted to a developing country (Cuba) with limited research resources and access to sequencing technology. Consequently, obtaining genomic data from a non-hybrid wild caught specimen was limited to the most accessible sequencing technology available at the time of collection. If and when more funds become available, the completeness and accuracy of the genome will be built upon using long-read sequencing technologies.

## Data Availability

The data described in this Data note can be freely and openly accessed on NCBI under BioProject PRJNA1005273, BioSamples SAMN36978604 [33]. The Supernova genome assembly can be found at NCBI under Accession No. JAVSML000000000 [34]. Please see Table 1 and references [32-34] for details and links to the data.

## Abbreviations

Kb: kilobases
Gb: gigabases
Mbp: million base pairs
bp: basepair
BUSCO: Benchmarking Universal Single-Copy Orthologs
IUCN: International Union for the Conservation of Nature
